# Quantification of regurgitation in mitral valve prolapse with four-dimensional flow cardiovascular magnetic resonance

**DOI:** 10.1186/s12968-021-00783-8

**Published:** 2021-07-08

**Authors:** Ricardo A. Spampinato, Cosima Jahnke, Gerard Crelier, Frank Lindemann, Florian Fahr, Monika Czaja-Ziolkowska, Franz Sieg, Elfriede Strotdrees, Gerhard Hindricks, Michael A. Borger, Ingo Paetsch

**Affiliations:** 1 Department of Cardiac Surgery, Heart Center Leipzig at University of Leipzig, Struempellstrasse 39, 04289 Leipzig, Germany; 2 Department of Cardiology and Electrophysiology, Heart Center Leipzig at University of Leipzig, Struempellstrasse 39, 04289 Leipzig, Germany; 3 Institute for Biomedical Engineering, University and ETH Zurich, Zurich, Switzerland

**Keywords:** Degenerative mitral regurgitation, Mitral valve prolapse, Quantification of mitral valve regurgitation, Four-dimensional, Cardiovascular magnetic resonance, Echocardiography

## Abstract

**Background:**

Four-dimensional cardiovascular magnetic resonance (CMR) flow assessment (4D flow) allows to derive volumetric quantitative parameters in mitral regurgitation (MR) using retrospective valve tracking. However, prior studies have been conducted in functional MR or in patients with congenital heart disease, thus, data regarding the usefulness of 4D flow CMR in case of a valve pathology like mitral valve prolapse (MVP) are scarce. This study aimed to evaluate the clinical utility of cine-guided valve segmentation of 4D flow CMR in assessment of MR in MVP when compared to standardized routine CMR and transthoracic echocardiography (TTE).

**Methods:**

Six healthy subjects and 54 patients (55 ± 16 years; 47 men) with MVP were studied. TTE severity grading used a multiparametric approach resulting in mild/mild-moderate (n = 12), moderate-severe (n = 12), and severe MR (n = 30). Regurgitant volume (RVol) and regurgitant fraction (RF) were also derived using standard volumetric CMR and 4D flow CMR datasets with direct measurement of regurgitant flow (4DF_direct_) and indirect calculation using the formula: mitral valve forward flow - left ventricular outflow tract stroke volume (4DF_indirect_).

**Results:**

There was moderate to strong correlation between methods (r = 0.59–0.84, p < 0.001), but TTE proximal isovelocity surface area (PISA) method showed higher RVol as compared with CMR techniques (PISA vs. CMR, mean difference of 15.8 ml [95% CI 9.9–21.6]; PISA vs. 4DF_indirect_, 17.2 ml [8.4–25.9]; PISA vs. 4DF_direct_, 27.9 ml [19.1–36.8]; p < 0.001). Only indirect CMR methods (CMR vs. 4DF_indirect_) showed moderate to substantial agreement (Lin’s coefficient 0.92–0.97) without significant bias (mean bias 1.05 ± 26 ml [− 50 to 52], p = 0.757). Intra- and inter-observer reliability were good to excellent for all methods (ICC 0.87–0.99), but with numerically lower coefficient of variation for indirect CMR methods (2.5 to 12%).

**Conclusions:**

In the assessment of patients with MR and MVP, cine-guided valve segmentation 4D flow CMR is feasible and comparable to standard CMR, but with lower RVol when TTE is used as reference. 4DF_indirect_ quantification has higher intra- and inter-technique agreement than 4DF_direct_ quantification and might be used as an adjunctive technique for cross-checking MR quantification in MVP.

**Supplementary Information:**

The online version contains supplementary material available at 10.1186/s12968-021-00783-8.

## Introduction

In past years, considerable advances in surgical treatment options of mitral regurgitation (MR) resulted in improved life expectancy, but prognosis and clinical decision making with regard to timing of surgery strongly depend on the accurate quantification of MR using cardiac imaging techniques [1].

Transthoracic multiparametric echocardiography (TTE) is at the forefront and widely recognized as the non-invasive standard of reference for assessment of MR [2] including proximal isovelocity surface area (PISA) determination with its typical methodological limitations (i.e. the reliance on geometric assumptions of a hemispheric flow convergence region (FCR), and Doppler measurement angle dependency). Hence, and particularly in borderline cases [3], a multimodality approach employing standard volumetric cardiovascular magnetic resonance (CMR) has been incorporated [4, 5].

In addition, time-resolved, three-dimensional (3D) full anatomic coverage with three-directional velocity-encoded phase contrast CMR, referred to as four-dimensional (4D) flow CMR has further broadened the diagnostic armamentarium [6] and has been proposed for quantification of blood flow volumes across the mitral valve. The potential benefit of 4D flow CMR in relation to the 2D CMR phase contrast (PC) technique, is the acquisition in one single examination of a “three-directional” velocity-encoding data-set. Permitting to place in a retrospective manner an analysis plane in any location perpendicular to blood flow, which may be critically important for quantify peak velocity and visualization of blood flow in cases of complex valve flow jets.

Previous 4D flow CMR studies using retrospective valve tracking or direct jet analysis, have been carried out in functional MR [7, 8], congenital heart disease [9], small patient groups [10], or in mixed patient populations [11, 12]. However, it can be speculated that these data cannot be generalized to patients with mitral valve prolapse (MVP) because concomitant MR may be challenging to assess due to geometrical asymmetry of the orifice area ("slit-like") and highly dynamic and eccentric MR jets. Thus, data on the clinical utility of 4D flow CMR in patients with MVP are still scarce. Therefore, we aimed to evaluate the value of cine-guided valve segmentation of 4D flow CMR in assessment of regurgitation in MVP when compared to standardized routine CMR and TTE.

## Material and methods

### Study population

Between June 2018 and December 2019, six healthy subjects and 58 patients with known MVP and sinus rhythm, who were referred to our outpatient clinic for follow up and gave written informed consent, were prospectively enrolled in an institutional review board–approved study. Exclusion criteria were previous valve surgery, concomitant aortic valve disease > grade I, intra-cardiac shunts, other known causes of cardiomyopathy, or typical contraindications for CMR imaging. The final study cohort consisted of 60 subjects (55 ± 16 years, 47 male); reasons for withdrawal from study were incapability to tolerate the supine position during the CMR examination (n = 1), incomplete 4D flow CMR dataset due to logistic reasons (n = 1) and low 4D flow CMR image quality due to multiple premature ventricular contractions during image data acquisition (n = 2).

Patient demographics and clinical data were recorded during initial presentation with TTE and CMR measurements carried out within a mean time frame of 6.5 h (mean 391 min, 95% CI: 125–658; range, 30–7320 min). Based on a TTE multiparametric approach [2] grading of MR severity resulted in three study groups: mild/mild-moderate (MR grade 1 + /2 + , n = 12), moderate-severe (MR grade 3 + , n = 12), and severe (MR grade 4 + , n = 30). In order to evaluate agreement with CMR quantification methods, regurgitant volume (RVol) and regurgitant fraction (RF) were also derived from standard volumetric CMR and 4D flow CMR.

### Standard echocardiography

TTE was performed using standard commercially available ultrasound machines (Vivid E95, General Electric Healthcare, Chicago, Illinois, USA; or Acuson SC2000 Prime, Siemens Healthineers, Erlangen, Germany) equipped with 2.25–4.25 MHz transducers. Evaluation of MVP was carried out by one cardiologist (RAS), expert in TTE and valvular heart diseases, blinded to the results of CMR exams. TTE were acquired using standard views and Doppler measurements were evaluated as the average of three cycles. Color flow Doppler interrogation of the MR jet was performed in multiple views. Vena contracta (VC) was measured in the modified parasternal long-axis view as the narrowest portion of the jet. PISA method, effective regurgitant orifice area (EROA), RVol, and RF were calculated as recommended [2]. Efforts were made to obtain a well-defined hemispheric FCR avoiding constraint. When necessary angle correction was advised to improve the accuracy. PISA radius was measured at the time of peak regurgitant velocity.

Following graduation scheme was used: mild (VC < 3 mm, EROA < 20 mm^2^, RVol < 30 ml, RF < 30%), mild-moderate (VC 3–6 mm, EROA 20–29 mm^2^, RVol 30–44 ml, RF 30–39%), moderate-severe (VC 3-6 mm, EROA 30–39 mm^2^, RVol 45–59 ml, RF 40–49%), and severe (VC ≥ 7 mm, EROA ≥ 40 mm^2^, RVol ≥ 60 ml, RF ≥ 50%). In case of discrepancies (in 13 of 54 MR cases) quantitative methods were conclusive.

### Standard volumetric CMR data acquisition and analysis

All CMR examinations were performed on dedicated 1.5 T CMR system (Ingenia, Philips Healthcare) equipped with Omega HP gradients (45 mT/m, 200 T/m/s) using a 28-element array coil with full in-coil signal digitalization. Image data acquisition adhered to current recommendations and image data analysis was carried out off-line [5] using IntelliSpacePortal analysis software (release 9.0.1, Philips Healthcare). All readers were fully blinded to clinical and TTE data. For cine CMR, balanced steady-state free precession (bSSFP) sequences with retrospective electrocardiographic (ECG) gating were used during repetitive breath-holding. All standard cardiac geometries were acquired (multiple, gapless short-axis slices covering the entire left ventricle (LV) and 2-, 3- and 4-chamber views). Reconstructed in-plane spatial resolution was 1.3 × 1.3 mm^2^ with a slice thickness of 8.0 mm; temporal resolution of cine bSSFP sequences was < 30 ms depending on heart rate. In addition, two-dimensional PC flow measurements were performed in the ascending aorta with the imaging plane 10 mm above the aortic valve and perpendicular to the flow direction; velocity encoding 200 cm/s was individually adapted if needed. Image data acquisition was ECG gated, with in-plane spatial resolution of 1.4 × 1.4 mm^2^, temporal resolution 35 phases per cardiac cycle, slice thickness 8 mm, and during a 12–15 s breath-hold. Through-plane phase-contrast derived measurements were: aortic stroke volume (AoSV), aortic systolic forward flow volume (AoFF), and aortic diastolic backward flow volume (AoBF). Cine short axis images were used to measure LV end-diastolic (LVEDV), LV end-systolic volume (LVESV), and stroke volume (SV). With standard volumetric CMR quantification of MR was performed indirectly. RVol was calculated by subtracting aortic systolic flow from LV SV. The RF was then calculated by dividing the RVol by the LV SV and expressed in percent: *RVol(ml)* = *LVSV–AoFF* and *RF(%)* = *(RVol / LVSV)* × *100* with severity graded as [13] mild (RF < 20%), mild-moderate (RF 20–29%), moderate-severe (RF 30–39%), and severe (RVol ≥ 55 ml, RF ≥ 40%).

### 4D flow CMR data acquisition and analysis

4D flow CMR acquisitions were obtained as the last sequence of each CMR study. Data were acquired by two cardiologists (CJ and IP) with over 20 years of experience in CMR imaging. The 4D flow CMR approach consisted in a LV long-axis alienated volume acquisition, planned in a four-chamber geometry. The number of slices has been adapted individually in order to ensure, from the two- and three-chamber views, a full coverage of left atrial, LV outflow tract (LVOT) and LV, encompassing the mitral valve during the whole cardiac cycle. Two different 4D flow CMR sequences were acquired with adapted velocity encoding to ensure no aliasing for the anterograde (stroke volume) and retrograde (RVol) mitral blood flow quantification. Reported acquisition nominal times for each 4D flow protocol were typically 5–10 min. The protocol used a field-of-view of 310 ± 15 mm with 70 ± 9 mm stack thickness reconstructed in 28 ± 3 slices of 2.5 mm thickness resulting in an acquired spatial resolution of 0.8–1.47 × 0.8–1.47 × 2.5 mm^3^. Flip angle was 10°, echo time/repetition time was 3.3/14 to 4.3/7.5 resulting in 22–56 ms (mean 38 ± 6 ms) temporal resolution.

4D flow CMR was supplemented with 2D cine acquisitions of LV two-, three-, and four-chamber views plus LVOT and mitral valve in-plane multi-slice 2D-cine geometries for high-resolution anatomical guidance, which were interpolated with the 4D flow datasets. In such a way that in-plane cine views as anatomical orientations, were available for mitral valve and LVOT segmentation ("cine-guided valve segmentation").

### Flow quantification

**Fig. 1:**
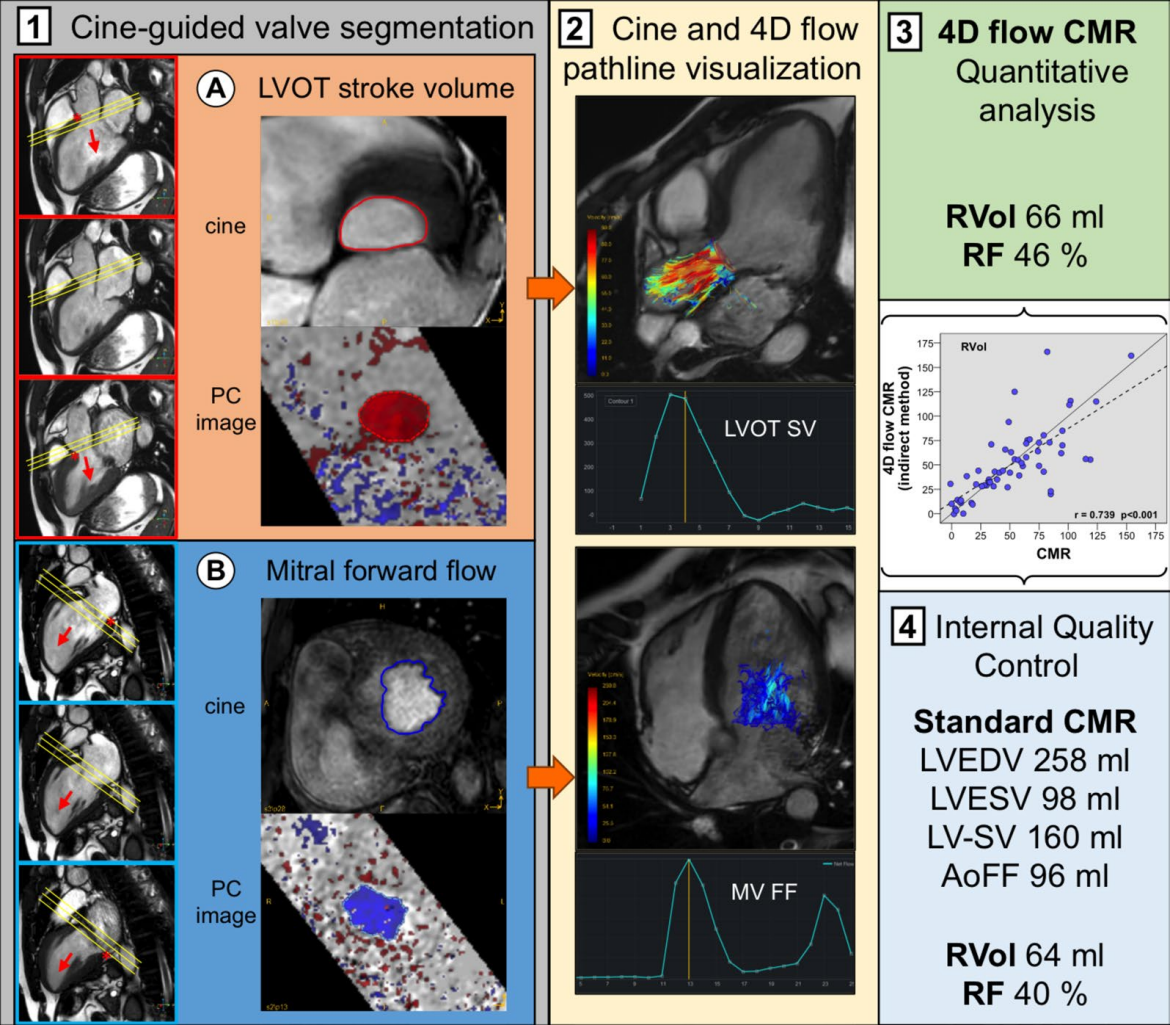
4D-flow cardiovascular magnetic resonance (CMR) imaging analysis. Step 1: planning of multiple, gapless short-axis slices covering the left ventricular (LV) outflow tract (LVOT) (upper red panels) and mitral valve (MV) (lower blue panels) through the entire cardiac cycle. Note the apical excursion of annular planes during systole (*). **A** and **B** cine-guided LVOT and mitral valve segmentation (upper panels) with corresponding interpolated 4D phase-contrast (PC) data (lower panels). Step 2: three-dimensional pathlines visualization emitted from LVOT and mitral valve planes superimposed on long-axis cines. Step 3: four-dimensional quantitative flow analysis and use of standard CMR indirect method for cross-checking (Step 4). An additional movie file shows this in more detail [see Additional file 2]. *LVOT* left ventricle outflow tract, *MV* mitral valve, *SV* stroke volume, *RVol and RF* regurgitant volume and fraction

Acquisition details are presented in Additional file 1 and depicted in Fig. 1. Post-acquisition, offline analysis was performed using commercially available custom software (GTFlow, GyroTools, Zurich, Switzerland). Subjects were analyzed in a blinded fashion (2 weeks apart from first TTE study) by a cardiologist (RAS) with 5 years clinical and research experience in CMR. Two readers for indirect (MCZ) and direct (FF) quantification obtained training and feedback in a series of 10 subjects. These feedback sessions were not included. Measurements were then repeated with 3 weeks between analyses to obtain intra- and inter-observer variability.

For indirect 4D flow CMR quantification (4DF_indirect_), the diastolic blood forward flow or stroke volume through the mitral valve (MV-SV_4D-flow_) and the systolic net blood flow or stroke volume through the LVOT (LVOT-SV_4D-flow_) were analyzed. Mitral valve regurgitant volume (RVol_indirect_) was calculated as the subtraction between MV-SV_4D-flow_ and LVOT-SV_4D-flow_, and the RF as follow: RF_indirect_ = (RVol_indirect_ / MV-SV_4D-flow_) × 100.

Additionally, the 4D flow CMR dataset with the higher venc was used to delineate the regurgitant jet for direct quantification (4DF_direct_) as previously described [14, 15]. A ROI was located using the PC image and the direct mitral valve regurgitant volume (RVol_direct_) derived. RF_direct_ = (RVol_direct_ / MV-SV_4D-flow_) × 100.

### Statistical analysis

Data are presented as mean (SD), median (25^th^–75th percentile), or frequency (percent) as appropriate. Statistical differences between groups were assessed using Student’s t-test for continuous variables or Fisher’s exact test for categorical variables. Multigroup comparisons of continuous variables were performed using an analysis of variance (ANOVA). Pearson correlation coefficient, Bland–Altman plots, and intraclass correlation coefficient (ICC) were used to assess correlations and agreements between methods. Then, Lin’s concordance correlation coefficient was calculated, to assess the concordance of continuous data with the following scale to describe the strength of agreement: > 0.99 indicates almost perfect agreement; 0.95–0.99, substantial agreement; 0.90–0.95, moderate agreement; and < 0.90, poor agreement. Additionally, rate of agreement for MR grading was evaluated by calculating *k*-statistics. Inter- and intra-observer coefficient of variation were determined as the deviation between (re)-measurements divided by the mean of both measurements. The mean absolute difference and the ICC were also determined. Finally, for internal validation, net forward flow through the mitral valve was considered the reference SV in the control group and through the pulmonary vale in 8 randomly selected cases of the MR group (validation subgroup, n = 14). Two-tailed p-values < 0.05 were considered statistically significant. Analyses were performed using SPSS (version 20, Statistical Package for the Social Sciences, International Business Machines, Inc., Armonk, New York, USA).

## Results

4D flow CMR analysis was successfully performed in 97% of subjects. 4DF_indirect_ quantification could be performed in all patients and controls (n = 60), while a 4DF_direct_ assessment in only 46 patients of the MR group (n = 54). A 2D-TTE derived PISA method could be obtained in all patients with MVP. There were no differences in blood pressure or heart rate values between CMR and TTE studies (132 ± 18 / 73 ± 11 vs. 133 ± 17 / 75 ± 11 mmHg, and 67 ± 11 vs. 68 ± 11 beats per minutes, p > 0.164). Post-processing times were shorter with 4DF_indirect_ method (indirect: 4.2 ± 0.7 min vs. direct: 6.4 ± 1.4 min; mean difference: 2.2 ± 1.3 min, p < 0.001).

Demographic and baseline patient characteristics are presented in Table 1. MR patients were older and more prone to have comorbidities than the control group. CMR and TTE characteristics are shown in Table 2. Patients with severe MR had higher TTE and CMR derived LVEDV and LV SV compared with MR grade 1 + /2 + and 3 + , but similar LVOT SV or forward SV values reflecting progressively higher RVol and RF values through the groups.

**Table 1:** Patient characteristics

	Controls (n = 6)	MR group (n = 54)
Age, years	31 ± 5	58 ± 14
Male, n (%)	5 (83)	42 (78)
BSA, m^2^	1.9 ± 0.2	1.9 ± 0.2
Coronary artery disease, n (%)	0	6 (11)
Hypertension, n (%)	0	39 (72)
Diabetes, n (%)	0	6 (11)
Dyslipidemia, n (%)	0	22 (41)
NYHA I**/**II**/**III-IV, n	6/0/0	22/19/13
Mitral valve prolapse		
Posterior leaflet, n	–	37
Anterior leaflet, n	–	3
Bileaflet (Barlow´s disease), n	–	14 (3)

*MR* mitral regurgitation, *BSA* body surface area, *NYHA* New York Heart Association Functional Classification. Unless otherwise specified, values are expressed as mean ± SD

**Table 2:** Multiparametric TTE classification of MR: TTE, standard, and 4D flow CMR values

	Control (6)	MR group (54)	MR Grade 1 + /2 + (12)	MR Grade 3 + (12)	MR Grade 4 + (30)
*Echocardiographic parameters*					
LVEDV, ml	133 ± 28	175 ± 67	133 ± 44^@^	140 ± 37^@^	206 ± 69
LVESV, ml	48 ± 9	58 ± 28	52 ± 21	44 ± 13	67 ± 33
LVEF, %	64 ± 4	67 ± 7	61 ± 6^#@^	68 ± 6	68 ± 6
LVOT SV, ml	80 ± 2	71 ± 16	73 ± 14	80 ± 13	67 ± 17
EROA, cm^2^ *	n.a	0.49 ± 0.28	0.16 ± 0.13^#@^	0.36 ± 0.09^@^	0.68 ± 0.22
RVol, ml *	n.a	73 ± 39	22 ± 16^#@^	54 ± 8^@^	101 ± 25
RF, % *	n.a	47 ± 18	21 ± 10^#@^	40 ± 4^@^	60 ± 8
Vena contracta, cm	n.a	0.62 ± 0.29	0.29 ± 0.15^#@^	0.60 ± 0.09^@^	0.76 ± 0.09
E wave, m/s	0.77 ± 0.21	1.36 ± 0.38^§^	0.92 ± 0.19^#@^	1.22 ± 0.16^@^	1.58 ± 0.31
*PW Doppler parameters*					
MV SV, ml	84 ± 3	139 ± 42^§^	96 ± 27^#@^	124 ± 16^@^	163 ± 38
RVol, ml	3 ± 3	69 ± 42^§^	22 ± 17^#@^	48 ± 13^@^	96 ± 36
RF, %	4 ± 3	45 ± 18^§^	21 ± 12^#@^	38 ± 7^@^	58 ± 11
*CMR parameters*					
LVEDV, ml	169 ± 24	220 ± 71^§^	172 ± 50^@^	186 ± 45^@^	254 ± 70
LVESV, ml	66 ± 12	85 ± 38^§^	70 ± 36	68 ± 25	98 ± 39
LV SV, ml	103 ± 14	135 ± 38^§^	102 ± 24^@^	117 ± 23^@^	156 ± 35
LVEF, %	63 ± 5	62 ± 7	61 ± 10	64 ± 7	62 ± 6
Aorta forward flow, ml	98 ± 12	78 ± 21^§^	82 ± 19	80 ± 16	76 ± 23
Aorta net flow (SV), ml	97 ± 11	74 ± 19^§^	77 ± 19	74 ± 12	72 ± 22
RVol, ml	5 ± 4	57 ± 34^§^	21 ± 16^#@^	37 ± 11^@^	80 ± 26
RF, %	4 ± 3	41 ± 17^§^	20 ± 13^#@^	33 ± 7^@^	53 ± 11
*4D flow CMR (indirect)*					
MV SV, ml	93 ± 16	124 ± 39	99 ± 25^@^	119 ± 23	135 ± 44
LVOT net flow (SV), ml	86 ± 15	68 ± 16^§^	73 ± 13	77 ± 9^@^	62 ± 17
RVol, ml **	7 ± 6	56 ± 35^§^	26 ± 16^#@^	43 ± 16^@^	73 ± 36
RF, % **	7 ± 6	42 ± 16^§^	24 ± 11^#@^	35 ± 6^@^	52 ± 13
*4D flow CMR (direct)*					
RVol, ml ***	n.a	51 ± 25	28 ± 12^@^	43 ± 15	59 ± 25
RF, % ***	n.a	40 ± 16	25 ± 9^@^	35 ± 9	46 ± 16

*TTE* transthoracic echocardiography, *MR* mitral valve regurgitation, *CMR* cardiac magnetic resonance, *LVEDV* left ventricular end-diastolic volume, *LVESV* left ventricular end-systolic volume, *LVEF* left ventricular ejection fraction, *LVOT* left ventricular outflow tract, *SV* stroke volume, *EROA* effective regurgitant orifice area, *RVol* regurgitant volume (MR), *RF* regurgitant fraction (MR), *PW* pulse-wave, *MV* mitral valve. Values are expressed as mean ± SD. *2D-PISA method (n = 54). **4D flow indirect (n = 60) and ***direct (n = 46) method. Differences reached statistical significance with: § control, # group “MR grade 3 + ”, and @ group “MR grade 4 + ”

### Agreement between methods

**Fig. 2:**
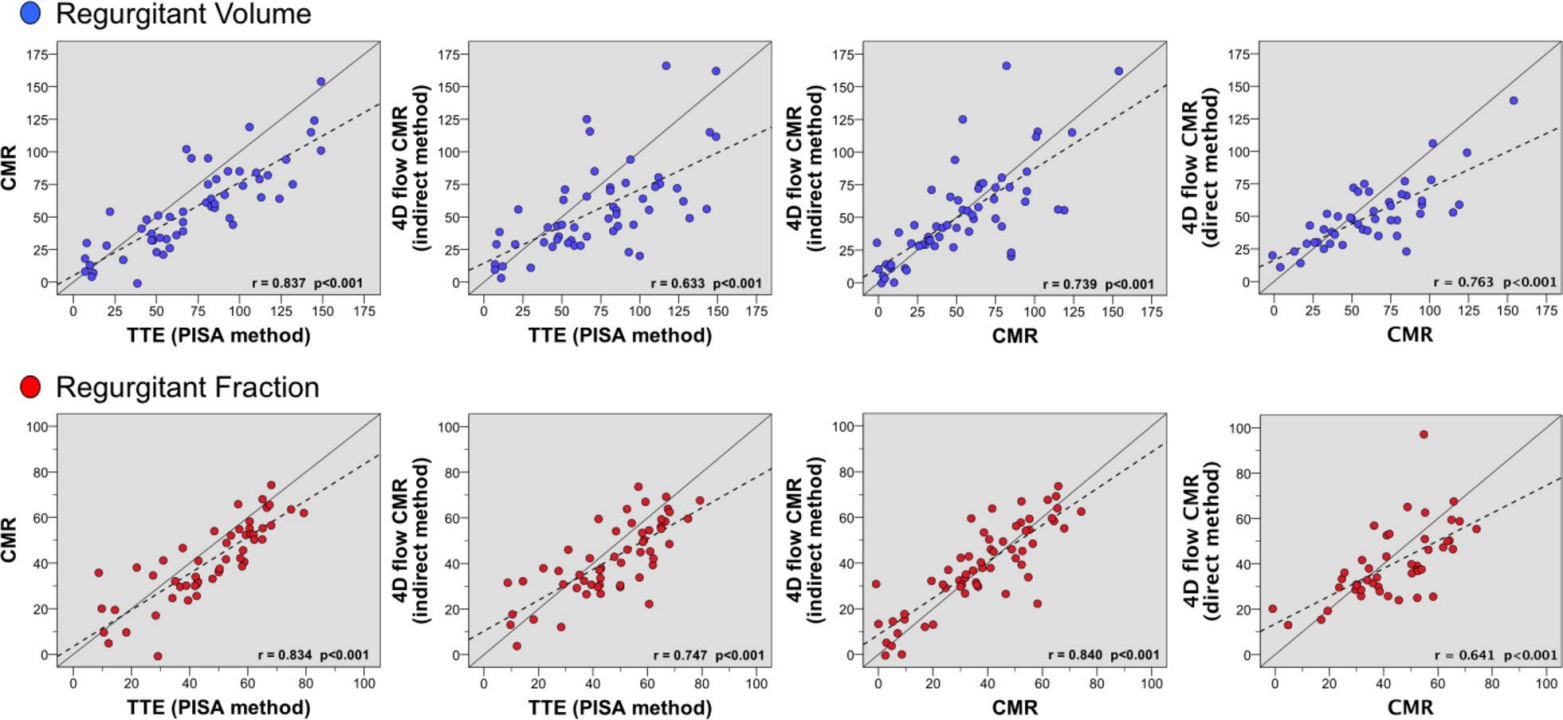
Correlation. For regurgitant volume (RVol) (blue dots) and regurgitant fraction (RF) (red dots) between 2D-transthoracic echocardiographic (TTE) flow convergence method (PISA), standard cardiovascular magnetic resonance (CMR), and 4D flow CMR indirect and direct methods. Dashed line indicates linear regression and solid line, line of identity. Pearson correlations (r) are showed

**Fig. 3:**
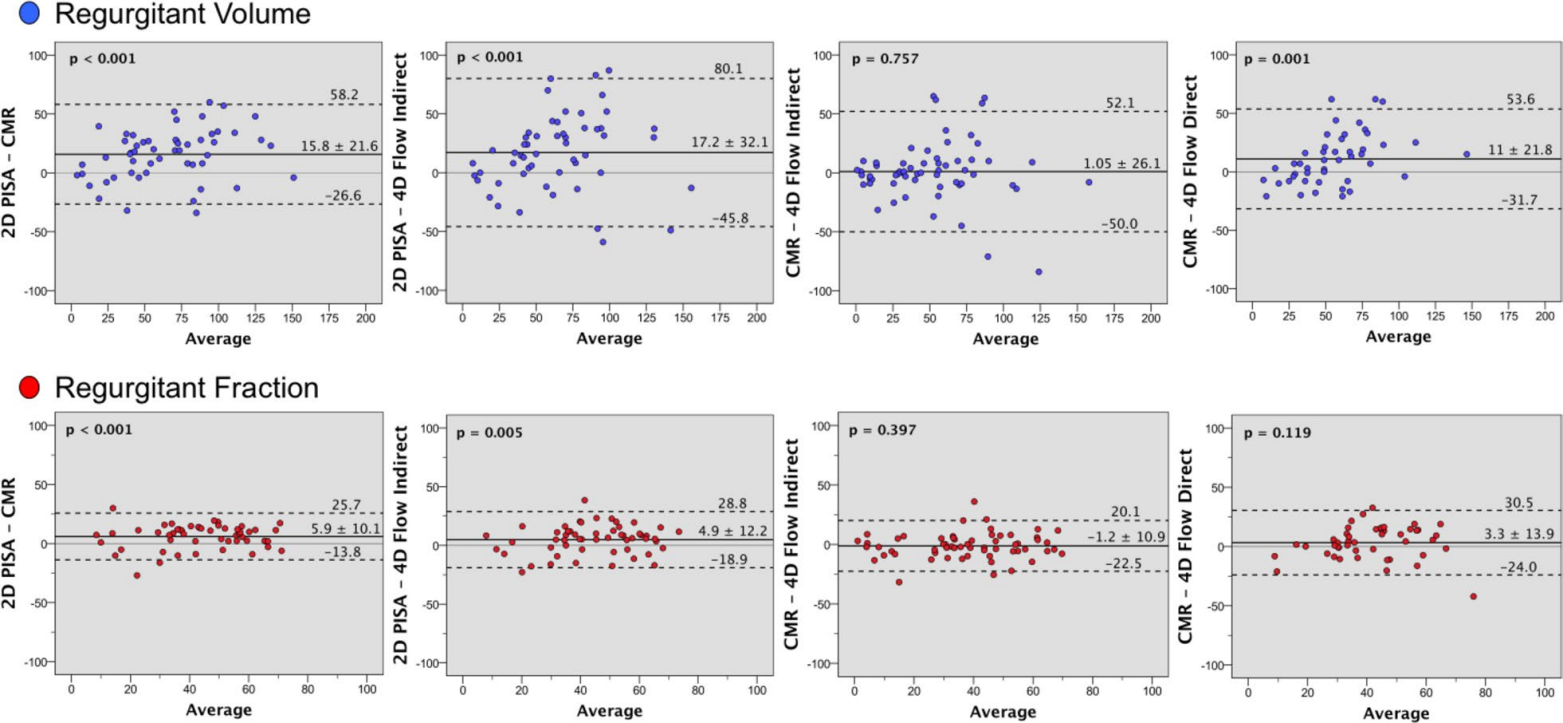
Bland–Altman plots. Agreement of measurements of regurgitant volume (blue dots) and fraction (red dots) by 2D-echocardiographic flow convergence method (2D-PISA), standard cardiac magnetic resonance (CMR), and 4D flow CMR indirect and direct methods, in patients with mitral valve prolapse

Overall the RVol and RF measurements assessed by PISA, CMR, and 4D flow methods showed moderate to strong correlation (Fig. 2). Nevertheless, PISA derived values were significantly different from standard and 4D flow CMR methods, with higher RVol and RF (Table 3 and Fig. 3).

**Table 3:** Comparison of regurgitant volume (RVol) and regurgitant fraction (RF) between methods

		Pearson (r)	Mean difference (95%CI)	P value	Absolute mean difference (95%CI)	Kappa^#^	ICC
2D-PISA vs. CMR	RVol	0.837	15.8 (9.9 to 21.6)	< 0.001	22.1 (18 to 26.1)		0.905
	RF	0.834	5.9 (3.2 to 8.7)	< 0.001	9.9 (8.3 to 11.6)	0.571*	0.909
2D-PISA vs. 4DF_ind_	RVol	0.633	17.2 (8.4 to 25.9)	< 0.001	28.8 (22.8 to 34.8)		0.772
	RF	0.747	4.9 (1.6 to 8.2)	0.005	10.9 (8.9 to 12.9)	0.510*	0.853
2D-PISA vs. 4DF_dir_	RVol	0.586	27.9 (19.1 to 36.8)	< 0.001	33 (25.9 to 40.1)		0.703
	RF	0.511	10.1 (5.4 to 14.7)	< 0.001	15.4 (12.3 to 18.5)	0.276*	0.676
CMR vs. 4DF_ind_	RVol	0.739	1.1 (− 5.7 to 7.8)	0.757	16.6 (11.4 to 21.8)		0.850
	RF	0.840	− 1.2 (− 4.0 to 1.6)	0.397	7.9 (6.0 to 9.9)	0.542	0.913
CMR vs. 4DF_dir_	RVol	0.763	11 (4.5 to 17.4)	0.001	18.5 (13.8 to 23.2)		0.842
	RF	0.641	3.3 (− 0.9 to 7.4)	0.119	10.7 (7.9 to 13.5)	0.383	0.780
4DF_ind_ vs. 4DF_dir_	RVol	0.764	9.2 (2.4 to 16)	0.009	16.3 (10.8 to 21.8)		0.834
	RF	0.472	4.4 (− 0.4 to 9.3)	0.069	11.8 (8.2 to 15.3)	0.277	0.641

*2D-PISA* 2-dimensional transthoracic echocardiography derived proximal isovelocity surface area, *CMR* standard cardiac magnetic resonance, *4DF*_*ind*_ 4-dimensional flow CMR *indirect* method, *4DF*_*dir*_ 4-dimensional flow CMR *direct* method, *ICC* intraclass correlation coefficient, *95%CI* 95% confidence interval. # Mitral regurgitation severity grading agreement between methods (*compared to integrative echocardiographic multi-parametric approach)

Bland–Altman plots demonstrated narrower limits of agreements for both indirect CMR methods. The small mean differences between 4DF_indirect_ and CMR derived RVol and RF were not significant (1.1 ml and 1.2%, respectively). In addition, calculation of Lin’s concordance correlation coefficients confirmed moderate to substantial agreement between standard CMR and 4DF_indirect_ for the assessment of RVol and RF, respectively. All other comparisons showed only poor agreement (Table 4). Moreover, agreement in grading MR severity was higher between standard CMR and 4DF_indirect_ than 4DF_direct_ (moderate, kappa = 0.542 vs. fair, kappa = 0.383; p < 0.001); with 39 of 54 patients (72%) having the same MR grade with the 4DF_indirect_ method. All controls were classified as none/trace MR in both indirect CMR methods. Finally, compared with an integrative TTE multiparametric approach, MR severity was reclassified more than one grade scale in 5 patients with the 4DF_direct_ method, in only 1 patient with the 4DF_indirect_ method, and in no patient with the standard CMR method, with consequently better *kappa*-statistics (Table 3).

**Table 4:** Lin’s concordance correlation coefficients to assess agreement between methods

		Lin's concordance correlation coefficient	95%CI
2D-PISA vs. CMR	RVol	0.851	(0.610 to 0.947)
	RF	0.852	(0.589 to 0.952)
2D-PISA vs. 4DF_ind_	RVol	0.841	(0.586 to 0.944)
	RF	0.865	(0.637 to 0.954)
2D-PISA vs. 4DF_dir_	RVol	0.538	(0.047 to 0.820)
	RF	0.331	(− 0.279 to 0.751)
CMR vs. 4DF_ind_	RVol	0.971	(0.924 to 0.989)
	RF	0.922	(0.793 to 0.972)
CMR vs. 4DF_dir_	RVol	0.862	(0.628 to 0.953)
	RF	0.596	(0.054 to 0.867)
4DF_ind_ vs. 4DF_dir_	RVol	0.670	(0.506 to 0.787)
	RF	0.448	(0.151 to 0.671)

*2D-PISA* 2-dimensional transthoracic echocardiography derived proximal isovelocity surface area, *CMR* standard cardiac magnetic resonance, *4DF*_*ind*_ 4-dimensional flow CMR indirect method, *4DF*_*dir*_ 4-dimensional flow CMR *direct* method, *95%CI* 95% confidence interval

When comparing the two 4D flow methods, there was a strong correlation assessing RVol (r = 0.764, p < 0.001), but only a weak to moderate correlation (r = 0.472, p < 0.001) for the RF values. 4DF_indirect_ method derived RVol values were statistically higher than those derived from 4DF_direct_ method, with a mean difference of 9.2 ml (95% CI 2.4 to 16.0, p = 0.009), but there were no statistical differences in RF values (4.4% [95% CI -0.4 to 9.3, p = 0.069]).

### Consistency and reproducibility of 4D flow quantification

In the validation subgroup (n = 14), the mean difference of 3.1 ml (95% CI, − 8.8 to 2.6; p = 0.259) between net forward flow volume through the LVOT (75.4 ± 16.5 ml) and the reference valve (78.5 ± 18.8 ml) was not significant (internal reference). Moreover, the lower values of the net forward flow volume through the LVOT by 4D flow compared to the 2D PC flow measurements (external reference) were also not significant (mean difference: − 7 ml, 95% CI, − 17 to 3.2; p = 0.162).

Reproducibility was tested in all subjects. The intra-observer coefficients of variation (CV) for analysis of MV-SV_4D-flow_ and LVOT-SV_4D-flow_ by 4DF_indirect_ method were 6.5 ± 5% and 4.6 ± 4.9%, with a mean difference of 1 ml (95% CI, − 3.8 to 1.9; p = 0.513) and 1.1 ml (− 2.3 to 0.2; p = 0.099), an absolute mean difference of 8.2 ml (6.2 to 10.2) and 3.3 ml (2.3 to 4.3), and excellent ICC of 0.982 (0.970–0.989; p < 0.001) and 0.979 (0.964–0.987; p < 0.001), respectively. The inter-observer CV were 11.9 ± 8% and 10.4 ± 10%, with a mean difference of 0.8 ml (95% CI, − 4 to 5.6; p = 0.735) and 1 ml (− 1.3 to 3.3; p = 0.390), an absolute mean difference of 14.7 ml (11.8 to 17.6) and 6.7 ml (5.2 to 8.3), and excellent ICC of 0.938 (0.896–0.963; p < 0.001) and 0.919 (0.865–0.952; p < 0.001), respectively.

The intra-observer CV for RVol quantification by 4DF_direct_ method was 16.6 ± 12%, with a mean difference of 2.7 ml (95% CI, − 5.3 to − 0.1; p = 0.041), an absolute mean difference of 7.5 ml (5.9 to 9.0), and an excellent ICC of 0.971 (0.948–0.984; p < 0.001). The inter-observer CV was 32 ± 21%, with a mean difference of 3.1 ml (95% CI, − 8.6 to 2.3; p = 0.257), an absolute mean difference of 15.2 ml (12.1 to 18.3), and a good ICC of 0.868 (0.762–0.927; p < 0.001).

Finally, inter- and intra-observer variability by 2D-PISA and standard CMR methods are also summarized in Table 5.

**Table 5:** Intra- and inter-observer measurement variability

		Bland–Altman*	ICC^†^	CV, %
*4D flow CMR indirect method*				
Intra-observer	MV SV	− 1.0 (21 to − 23)	0.982	7
	LVOT SV	− 1.1 (9 to − 11)	0.979	5
Inter-observer	MV SV	0.8 (37 to − 36)	0.938	12
	LVOT SV	1.0 (19 to − 17)	0.919	10
*4D flow CMR direct method*				
Intra-observer	MV RVol	− 2.7 (14 to − 20)	0.971	17
Inter-observer	MV RVol	− 3.1 (33 to − 39)	0.868	32
*Standard CMR method*				
Intra-observer	LV SV	1.5 (8 to − 5)	0.998	2.5
	Ao FF	− 0.1 (3 to − 3)	0.999	1.5
Inter-observer	LV SV	2.7 (19 to − 13)	0.989	6
	Ao FF	0.5 (10 to − 9)	0.986	5
*2D-PISA method*				
Intra-observer	MV RVol	2.1 (20 to − 16)	0.986	12
Inter-observer	MV RVol	3.2 (34 to − 28)	0.959	15

*4D Flow CMR* time-resolved, three-dimensional anatomic coverage, three-directional velocity-encoded phase contrast CMR, *MV* mitral valve, *LVOT* left ventricle outflow tract, *SV* stroke volume, *RVol* regurgitant volume, *LV* left ventricle, *Ao FF* aortic forward flow, *2D-PISA*, proximal isovelocity surface area, *ICC* intraclass correlation coefficient, *CV* coefficient of variation. *Mean difference (2-sided 95% confidence limits of agreement). † All P < 0.001

## Discussion

The present prospective study aimed to evaluate the clinical utility of cine-guided valve segmentation of 4D flow CMR for the assessment of MR in MVP in direct comparison to standard CMR and TTE. The main findings can be summarized as follows: (1) in patients with MVP, quantification of RVol and RF by 4D flow CMR was feasible and reproducible; (2) strong correlation was observed between standard CMR and 4DF_indirect_ methods, with no significant bias; conversely, 4DF_direct_ significantly underestimated RVol as compared with all other methods; (3) 4DF_indirect_ showed better intra- and inter-technique agreement and reliability than 4DF_direct_ method; and (4) despite moderate to strong correlation between methods, PISA method systematically showed higher RVol and RF as compared with CMR techniques.

Recently 4D flow CMR was introduced and validated in vitro with phantom models [11], and first clinical experiences with healthy subjects and patients with MR demonstrated its feasibility. However, previous studies have been performed in functional MR [7, 8], congenital heart disease [8, 15], small study groups [10, 16], or in rather mixed patient populations [9, 11, 12, 16]. In the present study we investigated the clinical utility of 4D flow CMR in a homogeneous group of MVP patients. Quantification of regurgitation in MVP is particularly challenging since extensive changes in valve geometry often lead to adherent or eccentric jets. Thus, volumetric measurement techniques may be preferable in MVP since regurgitant volume and fraction can be derived without any assumptions about hemodynamic or shape and are generally not or only minimally affected by the direction of the MR jet or geometry of the mitral orifice. First clinical studies using 4D flow CMR for direct assessment of both atrioventricular valves demonstrated excellent agreement between SV through the valve of interest and ascending aorta [11], or between all four valves [12]. The present study data demonstrated that 4DF_indirect_ is highly reproducible with higher concordance to standard CMR than 4DF_direct_. Recently, Fidock and colleagues [16] reported similar findings for primary MR. Nevertheless, they analyzed a small subgroup (n = 12), provided no direct comparison with TTE, and reported a rather low RVol (mean 28.6 ± 2.5 ml). In the present study the higher mean RVol of 57 ± 34 ml indicates that a substantial number of patients with high grade MR were included which will facilitate generalizability and applicability of our results in a clinical routine setting.

In the present study, 4DF_direct_ quantification could not be performed in 8 patients of the MVP group (n = 54) due to difficulties to delineate the cross-sectional area of the MR jet due to poor visualization (n = 4), excessive aliasing (n = 1), or poor contrast between blood pool and the regurgitant Jet (n = 3). Similarly, in a cohort of 44 children with congenital heart disease and atrioventricular valve regurgitation, Jacobs and colleagues reported that six patients (9%) were excluded due to concerns related to image quality including excessive aliasing on 4D flow (n = 1), movement during 4D flow acquisitions (n = 2), and poor visualization of the regurgitant jets (n = 3). Nevertheless, they reported a better concordance between standard CMR and 4D flow direct measurement of the regurgitant jet, as compared with an annular inflow indirect method, but with slightly superior performance in subjects with tricuspid regurgitation compared to those with MR [15]. Moreover and in accordance with our results, Feneis et al., in a cohort of 21 adults with tricuspid regurgitation (TR) and MR, showed that direct method was better for the assessment of TR, and the indirect method had a better diagnostic performance for the MR [10]. As mentioned by the authors, this could be explained because TR jets tend to be more central and laminar as compared to MR jets, which are more prone to be multiple, eccentric, and dynamic with systolic angulation change [14]. Which makes it challenging even with the use of retrospective valve tracking. This might explain the systematically lower values of RVol by 4DF_direct_ method observed in our study. Moreover, as previously showed [17], in patients with prominent bileaflet MVP, the volumetric CMR method may underestimate the LVESV as it only considers the volume located between the apex and the mitral annulus, and neglects the volume that is contained within the prolapsed mitral leaflets at end systole (prolapsed volume). Which may lead to an overestimation of the RVol. In our cohort we identified 14 patients with bileaflet MVP. In this subgroup, the mean prolapsed volume was 8.7 ± 4.8 ml. The corrected RVol tacking into account this prolapsed volume, as proposed from Vincenti et al., was accordingly significantly lower than the uncorrected RVol (30.4 ± 20.4 ml vs 39 ± 21.2 ml, p < 0.001). Notwithstanding, in this subgroup of patients, we observed no significant differences between uncorrected RVol by CMR and RVol by 4D flow (CMR: 39 ± 21 ml, 4DF_indirect_: 42 ± 32 ml, and 4DF_direct_: 37 ± 17 ml, p > 0.68). It might be speculated that measuring the anterograde mitral blood flow (MV-SV_4D-flow_) slightly above (ventricular side) or at the level of the mitral valve annulus may not discriminate between prolapsed volume and RVol. Nevertheless, retrograde mitral blood flow (RVol_direct_) was measured positioning the plane on the atrial side of the MV, and still the RVol by 4DF_direct_ was significantly higher than the corrected RVol (n = 12; 37 ± 17 ml vs 28 ± 18 ml, p = 0.008). Difficulties in identifying the contour of the cross-sectional regurgitant jet area using the 4D flow PC images may have contributed to overestimation of the RVol by 4DF_direct_ compared to corrected RVol, possibly due to applying slightly bigger ROI´s areas. However, the lack of a subgroup of patients with no MR and bileaflet MVP made the evaluation of this hypothesis not possible. Therefore, future studies will have to appraise this issue.

In addition, cine-guided valve segmentation provided better contrast of the borders of the mitral valve as compared with the contouring of the regurgitant jet in the 4D flow PC images. Aforementioned technical difficulties may also explain the observed longer post-processing time and higher inter-observer variability of the 4DF_direct_ method (CV: 32% vs. 12%). Kamphuis et al. [9] demonstrated a shorter analysis time to quantify the flow over all 4 valves for automated versus manual tracking (14 min vs. 25 min) and reported an excellent reproducibility for the measurement of mitral valve flow (intra-observer, CV 5.2%; inter-observer, 5.6%). Additionally, Jacobs et al. [15], reported excellent post-processing times for assessment of atrioventricular valve regurgitation, using a semi-automated 4D flow CMR algorithm (indirect method 3.7 ± 1.9 min vs. direct method 3.1 ± 2.1 min, p = 0.114).

Even though, we still observed broad limits of agreement between standard CMR and 4DF_indirect_ methods, the Lin´s coefficient showed moderate to substantial agreement and there were no systematic bias. These widespread limits of agreement may be expected to be more pronounced in the groups with MR 3 + /4 + , where the impact in grading of regurgitation should be less decisive. Indeed, consistency in grading MR severity between standard CMR and 4DF_indirect_ was clinically acceptable (kappa = 0.542; p < 0.001); with 75% of subjects having the same MR grade and 22% with only one scale misclassification. Only 2 of 60 subjects with MR 4 + by standard CMR were reclassified as MR 2 + by 4DF_indirect_. Importantly, all controls were classified as none/trace MR with both methods and no MR 1 + was reclassified as severe. Which, is a real scenario in the daily clinical practice. Other studies have showed similar limits of agreement when comparing two different methods of MR quantification [15, 18]. Calkoen et al. [14] found narrower limits of agreement between standard CMR and 4D flow (− 12 to 20 ml), but they included a cohort with a mean RVol of 11 ± 6 ml (vs. 57 ± 34 ml in our study).

Finally, we observed higher values of RVol and RF by TTE PISA method as compared with CMR techniques. There is some data suggesting that PISA method might overestimate the RVol in organic MR when compared with CMR [18, 19]. In accordance with our study, Uretsky et al. [18] observed similar bias between TTE and CMR (mean difference of 16 ml with wide limits of agreement: -38 to 70 ml). This might be in part secondary to difficulties in contouring the dynamic FCR. Thavendiranathan et al. [20] compared the diagnostic accuracy of 3D-PISA method in assessing functional MR using the peak 3D-PISA and the integrated 3D-PISA derived RVol (calculated for each systolic frame) taking into account the dynamic of the MR. They showed that compared with CMR derived RVol (33 ± 22 ml), the integrated 3D-PISA derived RVol (34 ± 26 ml) was not significantly different; however, the peak 3D-PISA derived RVol was higher (48 ± 27 ml).

In the present study, 4DF_indirect_ method performed comparably to standard 2D CMR, but with the need of additional acquisition and post-processing time. Nonetheless, 4D flow CMR may exhibit some potential translational outlooks and advantages. It permits to map out retrospectively the velocities and flow patterns throughout the whole heart, which may give a much more complete picture of the heart valve pathology, not only due to a 3D visualization of the regurgitant jet, but also because it could permit to derive advanced hemodynamic metrics (i.e. shear stress, turbulent kinetic energy, flow vorticity and helicity) which may help to better understand the pathophysiology and its relationship with clinical outcomes. Moreover, development of acceleration techniques, improved automated methods, and emerging machine learning algorithms, may improve 4D flow CMR techniques, allowing analysis of the entire cardiac hemodynamics in a retrospective manner for cross-checking quantification in case of uncertainty.

Future studies will have to validate our findings, and appraise the potential correlation of 4D flow CMR and of its derived advanced hemodynamic parameters with clinical outcomes in different scenarios, which could be of value in understanding the pathophysiology and progression to symptoms and adverse events in MVP patients.

### Study limitations

Several limitations apply to our study. For speed-up of image data acquisition and respiratory motion compensation, 4D flow CMR utilized the combination of parallel imaging with free-breathing, navigator based scanning. While this may represent a potential source of breathing motion-related artifacts or distortion of the three-directional velocity encoding data, we found in accordance with previously published data, a high rate of artifact-free, diagnostic image quality datasets (97%) and an excellent agreement between CMR techniques [11, 15].

Moreover, 2D cine of the valves were acquired on breath hold, while 4D flow was performed with free breathing. The use of such cine-guided valve segmentation relies on a combination of two different CMR techniques and may result in spatial misalignment between cine scans and 4D flow dataset, increasing the potential for flow measurement errors. However, after excluding major misalignment, we obtained accurate and reproducible data. On the other hand, previous CMR studies with 2D flow has been carried out using the older free-breathing flow sequences, which were recommended for accurate flow measurement due to lower background offset error and better resolution [21]. Notwithstanding, with breath-hold sequences using new aceleration techniques, we obtained excellent in-plane spatial and temporal resolution with acceptable consitency with the net forward 4D flow volume measurements in a validation subgroup (n = 14).

In conventional CMR, cardiac motion causes target structures to move in and out of the image plane. This through-plane motion of the valve plane causes systematic errors in the measurement of flow, which can lead to underestimation of RVol. Exacerbated by factors like vigorous longitudinal contraction of the LV (common in severe MR). These limitations may be overcome by the use of prospective slice tracking flow CMR sequences [22], but this involves complex software programming and it is not widely available so far. These concerns have resulted in an interest in the use of 4D flow CMR sequences with retrospective valve tracking. We proposed a cine-guided valve segmentation to at least partially overcome the through-plane motion of the mitral valve and LVOT planes. These sequences are widely available and may improve the anatomical display and delineation of the region of interest, due to the higher contrast between blood pool and surrounding structures of the balanced steady state free-precession and spoiled gradient echo sequences compared with the magnitude (anatomical) images of the 4D flow datasets. Nevertheless, they may still have introduced systematic errors in the measurement of the RVol. Despite this limitation, 4DF_indirect_ showed reasonable agreement with standard volumetric CMR quantification providing an alternative method for retrospective internal validation.

Finally, and as noted by other investigators, post-contrast 4D flow CMR data acquisition may benefit from the enhanced signal-to-noise and velocity-to-noise ratio as well as improved contrast between blood and surrounding tissue [23], which could increase the conspicuity of the MR jet on 4DF_direct_ jet analysis. Further investigation regarding the potential benefit of post-contrast 4D flow CMR is warranted.

## Conclusions

Present study demonstrated that cine-guided valve segmentation 4D flow CMR for the assessment of MR in MVP is feasible and may enable comparable evaluation to standard volumetric calculation CMR, but with lower RVol when TTE is used as reference. 4DF_indirect_ method has higher intra- and inter-technique agreement than 4DF_direct_ method and might be used as an adjunctive technique for cross-checking MR quantification in MVP.

## Supplementary Information

**Additional file 1.** 4D flow CMR Acquisition details.

**Additional file 2:** Movie file: 4D flow CMR indirect method: scene #1: cine-guided mitral valve and LVOT segmentation. scene #2: cine and 4D flow pathline visualization. scene #3: mitral valve and LVOT stroke volume measurement. 4D flow CMR direct method: scene #4: orthogonal stack planning. scene #5: eccentric regurgitant jet visualization.

## Data Availability

The datasets used and/or analyzed supporting the conclusions of the article are available from the corresponding author on reasonable request.

## Abbreviations

4D: Four-dimensional
AoBF: Aortic diastolic backward flow volume
AoFF: Aortic systolic forward flow volume
AoSV: Aortic stroke volume
bSSFP: Balanced steady state free precession
CMR: Cardiovascular magnetic resonance
ECG: Electrocardiogram
EROA: Effective regurgitant orifice area
FCR: Flow convergence region
LV: Left ventricle/left ventricular
LVEDV: Left ventricular end-diastolic volume
LVEF: Left ventricular ejection fraction
LVESV: Left ventricular end-systolic volume
LVOT: Left ventricular outflow tract
MR: Mitral regurgitation
MV: Mitral valve
MVP: Mitral valve prolapse
PC: Phase contrast
PISA: Proximal isovelocity surface area
RF: Regurgitant fraction
RVol: Regurgitant volume
SV: Stroke volume
TTE: Transthoracic echocardiography
VC: Vena contracta
