# Supplementary material for: Quantification of regurgitation in mitral valve prolapse with four-dimensional flow cardiovascular magnetic resonance
Source: J Cardiovasc Magn Reson. 2021 Jul 8;23:87. doi: 10.1186/s12968-021-00783-8 (PMC8265147; doi:10.1186/s12968-021-00783-8)
Supplement: Supplementary file 1 — Additional file 1. 4D flow CMR Acquisition details. [file 12968_2021_783_MOESM1_ESM.docx]

**Additional File 1**

**Appendix A**

**4D flow CMR Acquisition details:**

The employed flow-encoding scheme was Hardaman 4-point encoding. Anterograde mitral 4D flow CMR sequence used four-point three-directional velocity encoding with Venc between 150 cm/s and 250 cm/s per anticipated maximal velocity through the mitral valve according to pulsed Doppler echocardiography measures, and for retrograde mitral 4D flow CMR sequence with Venc between 500 cm/s and 650 cm/s per anticipated maximal velocity according to systemic blood pressure. Thus, efforts were made to avoid the use of a phase-unwrapping algorithm. Both sequences used retrospective gating with 25 phases reconstructed, obtained with free breathing and 3 signal averages taken to suppress breathing motion artifacts, and parallel imaging with sensitivity-encoding factor 2 for accelerated read-out. To further limit the total scan-time between 5–10 min, spatial resolution and SNR were reduced by acquiring a percent of ky and kz phase encoding lines (but not less than 65% × 65%). Commercially available concomitant gradient (Maxwell) and Eddy current correction was performed by scanner software. The protocol used an individually adjusted field-of-view of 310±15 mm with 70±9mm stack thickness reconstructed in 28±3 slices of 2.5 mm thickness resulting in an acquired spatial resolution of 0.8 - 1.47 × 0.8 - 1.47 × 2.5 mm^3^. Flip angle was 10°, echo time/repetition time was 3.3/14 to 4.3/7.5 resulting in 22–56 msec (mean 38±6 msec) temporal resolution.

Two-dimensional steady-state free-precession short axis cine acquisitions of left ventricular outflow tract and mitral valve were planned with in-plane geometry adapted from long axis planes. Three in-plane slices were acquired (the central one was located in mid portion of LVOT and between MV annulus and tip of opened leaflet) with slice thickness 8 mm, gap -1. For better anatomical depiction of the mitral valve with higher contrast between blood pool and mitral valve tissue, additional cine imaging of the mitral valve was performed using spoiled gradient echo (SGE) sequences with in-plane geometry adapted on a 4-, 2- and 3-chamber view (repetition time/echo time 5.2 ms/3.1 ms; 30 phases/cardiac cycle; slice thickness 8 mm; in-plane spatial resolution 1.0 x 1.0 mm; sensitivity-encoding factor 1.8; breath-hold duration per slice 8-9 seconds). Cine images were then fused and time-interpolated with 4D flow CMR phase-contrast images to improve the anatomical display and delineation of the region of interest (ROI).

All volunteers and patients, except in one patient, were scanned without the use of contrast agent.

**Flow quantification:**

First step was to verify the quality of 4D data set. As recommended (1) visual inspection of the magnitude and phase contrast images and exclusion of aliasing or phase wraps, background phase offsets (marked disruption of streamlines or pathlines), and other image artifacts were performed. Finally, check for substantial spatial misalignment between the cines and the 4D flow data was performed(2). When a clear lack of quality was observed, the data set was rated as unusable.

***Indirect measurement of mitral valve regurgitant volume. Mitral valve and left ventricular outflow tract stroke volume***.

The 4D flow CMR data set with the lowest Venc (150 to 250cm/s) was used. The volumetric coverage of 4D flow CMR offers retrospective positioning of planes for flow volume measurements at any location. Thus, the interpolated short-axis cines were used as geometrical boundaries in the flow volume calculation **(cine guided valve segmentation)**. The three slices of the short-axis were used to follow and delineate the ROI during the desired period of the cardiac cycle. Net flow was calculated as volume velocity from each frame multiplied by time step (seconds per frame).

The data set was rated as unusable in case of a non-visible mitral valve, failure to interpolate both datasets, or any other cause that not permit to obtain a clear mitral valve in-flow or LVOT out-flow curve.

For *indirect* MR quantification, the diastolic blood flow or stroke volume through the MV (MV-SV_4D-flow_) and the systolic net blood flow or stroke volume through the LVOT (LVOT-SV_4D-flow_) were analyzed. Finally, the MV regurgitant volume (RVol_indirect_) was calculated as the subtraction between MV-SV_4D-flow_ and LVOT-SV_4D-flow_, and the RF as follow: *RF_indirect_ = (RVol_indirect_ / MV-SV_4D-flow_) x 100*. Time needed for complete analysis was recorded. See also Additional file 2.

***Direct measurement of mitral valve regurgitant volume.***

Additionally, the 4D flow CMR dataset with the higher Venc (500 to 650cm/s) was used to delineate the regurgitant jet for *direct* quantification as previously described(3,4). Long-axis cine images were used to position a stack perpendicular to the Jet downstream slightly under the level of the coaptation defect (vena contracta). A ROI was then located using the PC image and the direct MV regurgitant volume (RVol_direct_) derived. *RF_direct_ = (RVol_direct_ / MV-SV_4D-flow_) x 100*. See also Additional file 2.

The data set was rated as unusable in case of multidirectional jets, extremely dynamic and direction changing jet, non-visible jet or vena contracta.

**References**

1. Dyverfeldt P, Bissell M, Barker AJ et al. 4D flow cardiovascular magnetic resonance consensus statement. J Cardiovasc Magn Reson 2015;17:72.

2. Garg P, Swift AJ, Zhong L et al. Assessment of mitral valve regurgitation by cardiovascular magnetic resonance imaging. Nat Rev Cardiol 2020;17:298-312.

3. Calkoen EE, Westenberg JJ, Kroft LJ et al. Characterization and quantification of dynamic eccentric regurgitation of the left atrioventricular valve after atrioventricular septal defect correction with 4D Flow cardiovascular magnetic resonance and retrospective valve tracking. J Cardiovasc Magn Reson 2015;17:18.

4. Jacobs K, Rigdon J, Chan F et al. Direct measurement of atrioventricular valve regurgitant jets using 4D flow cardiovascular magnetic resonance is accurate and reliable for children with congenital heart disease: a retrospective cohort study. J Cardiovasc Magn Reson 2020;22:33.
